# Dollo meets Bergmann: morphological evolution in secondary aquatic mammals

**DOI:** 10.1098/rspb.2023.1099

**Published:** 2023-07-12

**Authors:** B. M. Farina, S. Faurby, D. Silvestro

**Affiliations:** ^1^ Department of Biology, University of Fribourg, Fribourg, Switzerland; ^2^ Swiss Institute of Bioinformatics, Fribourg, Switzerland; ^3^ Department of Biological and Environmental Sciences, University of Gothenburg, Gothenburg, Sweden; ^4^ Gothenburg Global Biodiversity Centre, University of Gothenburg, Gothenburg, Sweden

**Keywords:** body size, irreversibility, macroevolution, mammals, terrestrial–aquatic transitions

## Abstract

Secondary transitions to aquatic environments are common among vertebrates, and aquatic lineages display several adaptations to this realm, some of which might make these transitions irreversible. At the same time, discussions about secondary transitions often focus only on the marine realm, comparing fully terrestrial with fully aquatic species. This, however, captures only a fraction of land-to-water transitions, and freshwater and semi-aquatic groups are often neglected in macroevolutionary studies. Here, we use phylogenetic comparative methods to unravel the evolution of different levels of aquatic adaptations across all extant mammals, testing if aquatic adaptations are irreversible and if they are related to relative body mass changes. We found irreversible adaptations consistent with Dollo's Law in lineages that rely strongly on aquatic environments, while weaker adaptations in semi-aquatic lineages, which still allow efficient terrestrial movement, are reversible. In lineages transitioning to aquatic realms, including semi-aquatic ones, we found a consistent trend towards an increased relative body mass and a significant association with a more carnivorous diet. We interpret these patterns as the result of thermoregulation constraints associated with the high thermal conductivity of water leading to body mass increase consistently with Bergmann's rule and to a prevalence of more nutritious diets.

## 1. Introduction

Evolutionary transitions to physically distinct realms are relatively rare in the history of life [1]. A transition from water to land is thought to have happened only once in vertebrates [1], while land-to-water transitions are more common [1,2]. Starting around the Permian–Triassic boundary, several lineages of tetrapods have independently evolved into fully aquatic forms, including several apex predators in the marine realm [3–8]. Independent transitions to aquatic environments in tetrapods occurred in a diverse range of clades such as ichthyosaurs, whales and penguins, which all independently converged to morphologies optimized for swimming [5,9,10].

Adapting to the constraints and opportunities presented by new realms requires several unique evolutionary innovations [5,11,12]. For instance, when transitioning from a terrestrial to a buoyant environment, different lineages independently evolved similar adaptations to be able to survive in the new habitat, such as streamlined body plans, dorsal nares and similar locomotory systems [5,12–15]. These secondary aquatic adaptations in tetrapods are often interpreted as examples of Dollo's Law, which predicts the irreversibility of the loss of complex characters [16]. Technically, these transitions are either irreversible or just extremely unlikely to be reversed, but for simplicity, we will refer to them hereafter as irreversible. For instance, the reduced legs in some marine tetrapods—and, consequently, their inability to walk—is thought to follow Dollo's Law [17,18].

Another morphological adaptation involved in secondary aquatic transitions in tetrapods is body size increase [14,19]. When transitioning to water, many secondary marine clades, such as whales and turtles, evolved large body sizes (e.g. [11,20–24]). However, the consistency of this pattern of body size increase linked to aquatic transitions across clades remains uncertain, partly because the numerous semi-aquatic freshwater lineages are often neglected. Different hypotheses have attempted to explain patterns of body mass and size over time. One of them, Bergmann's rule, describes a consistent pattern of increased body sizes in homeotherms associated with high latitudes or cold regions [25,26], a process likely be linked to lower relative heat loss in larger animals [27]. A similar pattern can be expected in aquatic lineages, as the large heat capacity of water increases the relative heat loss for aquatic animals compared to terrestrial ones [11,21].

In addition to changes in relative body mass, transitions to aquatic environments could lead to modifications of diet in tetrapods due to differences in resource availability or in metabolic requirements. Field metabolic rates have proven to be very hard to measure for fully aquatic animals but at least some studies have indicated increased metabolism in marine mammals, supporting this idea [28]. This is also supported in turtles, where fully terrestrial tortoises are mostly herbivorous while freshwater and marine species are more commonly omnivores or carnivores [29].

Discussions about the evolution of secondary aquatic vertebrates often focus on the most aquatic forms, and studies about mammalian secondary transitions and adaptations are no exception. These, however, represent a fraction of the lineages showing at least some adaptations to aquatic lifestyle, with most of the semi-aquatic clades living in freshwater [22,23]. Substantial work has been made on the wide diversity of aquatic adaptations (e.g. [2,11,20,24,30]). Yet, the full spectrum of freshwater or semi-aquatic lineages is often not included on secondary aquatic transitions (e.g. [1,2,11,12,30]; but see [31,32]) making it difficult to assess the generality of patterns associated with aquatic adaptation in mammalian and in tetrapod evolution.

Here, focusing on extant and recently extinct mammals, we evaluate the macroevolutionary processes leading to intermediate and full transitions to aquatic environments, testing to what extent such transitions conform to Dollo's Law. We additionally investigate the phenotypic and dietary changes associated with these transitions and their mode of evolution, focusing on body mass to test if shifts to aquatic environments are consistent with Bergmann's rule and if adaptations to aquatic lifestyle are more common in carnivores which could be expected based on metabolic constraints.

## 2. Methods

### (a) Dataset and aquatic classification

We compiled mammal taxonomic data and habitat information from the IUCN Red List database [22]. Body mass, as proxy for body size, and diet information (i.e. fraction of vertebrate, invertebrate and plant eaters) were obtained from the PHYLACINE database [33] and originally come from a large number of sources but primarily from Smith *et al*. [34], Faurby & Svenning [35], Wilman *et al.* [36] and Kissling *et al.* [37]. Additionally, we obtained phylogenetic information from the most recent and comprehensive phylogenetic tree of extant and recently extinct mammals [38]. Taxonomy was matched between the sources and only species present in the IUCN Red List database [22] were considered. Our final dataset included 5635 species of extant and recently extinct (as defined in the Red List) mammals.

We classified the adaptations to aquatic environments in mammal species into four categories, based on the literature [39–42]. Our classification is based on species aquatic adaptations, reflecting previous work [43], but without distinguishing between freshwater and marine environments. We classified the species into four categories:

A0—No aquatic adaptations (hereafter referred to as ‘terrestrial’). Most of these species are fully terrestrial, but this group also includes some species often classified as semi-aquatic. Specifically, it includes all species without distinct morphological adaptations to movement or feeding in water (e.g. giraffe (*Giraffa camelopardalis*), gorilla (*Gorilla* sp.), or elephants). This means that some species which spend substantial amount of time in water, like hippos (*Hippopotamus amphibius*), are coded as terrestrial in our coding scheme. In this category, we restricted the coding to physical rather than behavioural traits because many smaller mammals, including many rodents and insectivores, have never, or only very rarely, been observed alive, and including behavioural adaptations would bias our scoring so more studied species would be disproportionally likely to be coded as aquatic.
A1—Aquatic adaptation but fully moveable on land. This group contain species with some kind of morphological adaptation to aquatic lifestyle (such as interdigital webbing) but they do not have any problems moving on land and spend a considerable amount of time outside water, e.g. water shrew (*Neomys fodiens*), Iberian desman (*Galemys pyrenaicus*) or platypus (*Ornithorhynchus anatinus*).
A2—This group contains lineages with limited locomotion on land but still can be seen onshore regularly—Pinnipedea and the sea otter (*Enhydra lutris*).
A3—This group contains fully aquatic taxa lineages that never leave water—whales and sirenians.

Unlike for A0–1, we used both morphological and behavioural traits to differentiate the A2–3 lineages. These lineages, however, also show strong morphological modifications of their limbs separating them clearly from taxa in A1. Thus, even though A1 lineages include poorly studied species, particularly among rodents, their aquatic adaptations are substantially weaker than in the A2–3 lineages and there are no osteological indications that they should be inefficient for terrestrial movement.

### (b) Ancestral state inference and stochastic mapping

We modelled the evolution of aquatic adaptations across mammal lineages using phylogenetic comparative methods to infer the rates and mode of evolution, and the ancestral states [44]. We fitted seven Markov models—including reversible ones with all equal rates (ER; electronic supplementary material, figure S1a), with different but symmetric rates (SYM; electronic supplementary material, figure S1b), with all rates different (ARD; electronic supplementary material, figure S1g), and with transitions allowed only among adjacent states (ORD; electronic supplementary material, figure S1c). To assess whether there is evidence of reversals from aquatic to terrestrial adaptations, we included modified versions of the ORD model, implementing different degrees of irreversibility among states, where some of the transitions were only allowed from less to more aquatic but not the opposite (electronic supplementary material, figure S1d–f). We fitted the models in the maximum-likelihood framework implemented in the R [45] package *corHMM* [46] and replicated the analyses on a posterior sample of 100 trees (sampled randomly from the posterior distribution from Upham *et al*. [38]). We used the *maddfitz* method to infer the root state [47]. The best model was chosen based on Akaike information criterion corrected for finite sample size (AICc). We additionally ranked the models based on their relative probability approximated through Akaike weights [48].

To summarize the estimated transition rates across states while capturing model uncertainties, we performed model averaging based on the Akaike weights (AICc weights). Specifically, we summarized the estimated rate matrix across different models as the average weighted by the Akaike weight of each model. We replicated this across the 100 trees and calculated the mean rate matrix and its standard deviation. We also used stochastic character mapping to estimate the number of state transitions across 10 stochastic maps for each of the 100 trees using the function *makeSimmap()* from the R [45] package *corHMM* [46]. As model for the analysis, we used the best model matrix rate for each posterior tree. To summarize the number of transitions across the entire phylogeny, we used the function *countSimmap()* from *Phytools* [49], and calculated the median and credible intervals for the number of transitions. Using the same stochastic character maps, we summarized the empirical transition rates across 21 clades of mammals by dividing the estimated number of transitions within the clade by the total branch length of the clade. The 21 clades mostly reflected mammalian orders, but some monotypic or small clades were merged with their sister groups. Namely, we assumed Euarchonta clade, encompassing Scandentia + Dermoptera + Primates and Agreodontia clade, that includes Notoryctemorphia + Dasyuromorphia + Peramelemorphia. We did not compute the empirical transition rates for monotypic orders Microbiotheria and Tubulidentata.

In order to test for correlation between aquatic transitions and diet, we used the D-test [50] as implemented in the *Phytools* function *Dtest()* [49]. The D-test uses stochastic character mapping to quantify the strength and statistical significance of correlations among the states of two discrete traits [50]. We thus classified diet into three categories based on the fraction of invertebrates and vertebrates. Species with less than 20% of invertebrates and vertebrates in their diet were classified as herbivores; those with a range from 20 to 80% were considered omnivores; and above 80%, carnivores. The test was done using 100 stochastic maps to quantify the effect size (direction and strength of the correlation) running a total of 1000 posterior predictive simulations as a null distribution to estimate the statistical significance as predictive *p*-values [50,51].

### (c) Body mass evolution

We inferred body mass evolution across mammals to assess whether the transitions across different degrees of aquatic adaptations are associated with consistent relative changes in body mass. We used the Bayesian framework implemented in *fossilBM* [52], which implements algorithms that can sample evolutionary parameters and ancestral states efficiently even across large phylogenies, thus making it suitable for our dataset [52,53]. The method implements Brownian motion evolutionary models and allows evolutionary rates to vary across the phylogeny. The evolutionary rate quantifies the expected variance in the trait accumulated per time unit. The model also implements directional Brownian processes where a trend parameter quantifies the presence and directionality of consistent relative changes in the expected mean trait values as a linear function of time. Thus, a positive (negative) trend indicates a tendency towards larger (smaller) trait values through time, while a trend parameter equal to zero reduces the model to a neutral diffusion process. As with the evolutionary rates, *fossilBM* allows for variation in the trend parameter across clades [52]. Here, we extended the functionality of *fossilBM* to allow for the same estimated trend parameter to be shared among different lineages identified by a discrete trait mapped on the tree.

We implemented models that assume order-specific evolutionary rates and trait-dependent trends. To account for rate heterogeneity across lineages, we set up our model assuming an independent rate for each of the 21 clades described above and used also to infer empirical transition rates. In total, our analysis included 22 rate partitions, i.e. 21 clades + a background rate at the root and extending to the monotypic orders Microbiotheria and Tubulidentata. We also defined individual trend parameters associated with each degree of water adaptation. Our model therefore included three trend parameters (*μ*_A1_, *μ*_A2_, *μ*_A3_) associated with the three degrees of water adaptation (A1–A3), while we assumed neutral evolution for the terrestrial lineages (i.e. *μ*_A0_ = 0). We assumed these parameters to be shared across all lineages assigned to states A0–3, based on the ancestral state estimation described above.

We assigned a normal prior *N*(0, ***σ***) on the trend parameters so that the highest prior probability is assigned to the null assumption of a model of evolution with no trend (i.e. *m* = 0) [52]. We extended the model by treating ***σ*** as a free parameter, instead of fixing it to an arbitrary value. Thus, we sampled ***σ*** through the MCMC algorithm along with the other parameters (the ancestral states, the rates, and the trends) after assigning it an exponential hyperprior. This hierarchical modelling allows us to avoid setting an arbitrary value for ***σ***, while applying further regularization and reduce the risk of over-parametrization of the model (e.g. [51]). For comparison, we also ran the analyses with the default value ***σ*** = 0.1. We used a similar approach for the prior on the rate parameters. Here, we used an exponential prior Exp(*λ*) on the rates, where the parameter *λ* was itself considered as unknown, assigned an exponential hyperprior Exp(1), and sampled it through MCMC.

We additionally tested three simpler models. The first included a single rate parameter (thus removing rate heterogeneity among orders) and used a single shared trend parameter for all aquatic lineages (*μ*_A0_ = 0, *μ*_A1_ = *μ*_A2_ = *μ*_A3_). The second included rate variation but constrained trends, while the third assumed a constant rate but allowed for different trends across lineages based on their aquatic state.

We compared the results with a set of simulations aimed to assess the identifiability and robustness of the trend parameters. We simulated three separate scenarios using the mammal phylogenetic tree with our partition settings and simulating quantitative traits under different modes of evolution. The first scenario assumed neutral evolution across clades (i.e. no trend), while the true rate for each clade was randomly sampled from the respective posterior distribution resulting from the analyses of the empirical data. This scenario allowed us to test if the model correctly identifies the absence of trends. The second scenario assumed positive or negative trends, randomly sampled from a uniform distribution U(−0.1, 0.1) for each water-adapted category, and neutral evolution for terrestrial (A0) clades. Finally, we simulated a third scenario with different trends for each water-related category (randomly drawn as above), but also assuming a random trend for each A0 lineage. Scenarios two and three allowed us to assess whether the model can correctly identify true trends. All simulations were replicated across 100 posterior trees and analysed with and without hyperprior on *μ*.

## 3. Results

### (a) Secondary aquatic adaptations in mammals

**Figure 1.**
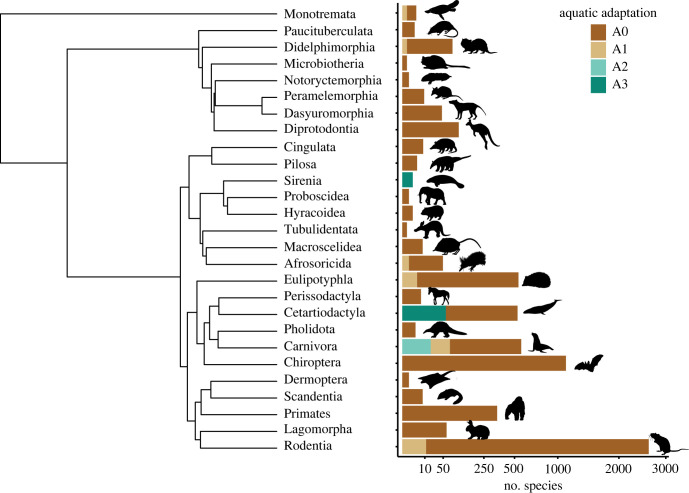
Summary of the mammalian phylogeny, grouped by orders, and showing how they were classified under our categorization. The scale for the number of species in the bar plot is log-transformed.

Among the 5635 collected species, 96.7% (5449 species) of them are fully terrestrial, while semi-aquatic and fully aquatic represent only 3.3% (186 species) of species and are distributed among eight different orders (figure 1; electronic supplementary material, table S1). Except for the platypus (*Ornithorhynchus anatinus*) within Monotremata and the water opossum (*Chironectes minimus*) within Didelphimorphia, both classified as A1, all aquatic adaptations are found within Placentalia (placental mammals). Semi-aquatic (A1) is seen in six orders—Monotremata (monotremes), Didelphimorphia (opossums), Afrosoricida (golden moles and tenrecs), Eulypotyphla (insectivores), Carnivora (carnivores) and Rodentia (rodents). Fully aquatic species (i.e. A2 and A3) are found in four lineages: pinnipeds (i.e. walrus, sea lions, fur seals and true seals) and the sea otter (*Enhydra lutris*), both within Carnivora; Sirenia (dugongs and manatees); and Cetacea (whales), within Cetartiodactyla.

**Figure 2.**
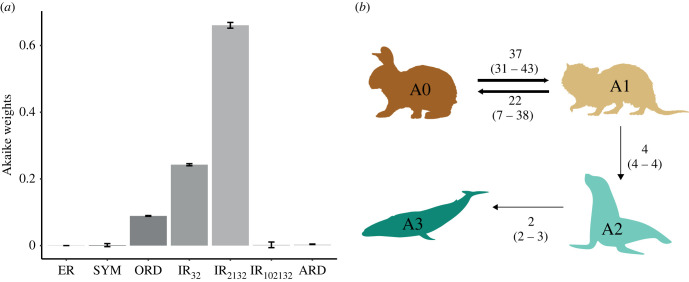
(*a*) Comparison between the different models using Akaike weights; (*b*) number of transitions (median and 95% credible intervals) between states calculated from stochastic mapping using the best model matrix.

Our analyses using phylogenetic comparative methods found strong statistical support for a scenario in which aquatic adaptations become irreversible after a threshold identified between the A1 and A2 categories. Among the seven tested models, the one with the highest support—with the lowest AICc score and a mean Akaike weight of 0.661—allowed for transitions between adjacent states with reversible transitions only between A0 and A1. This means that a fully terrestrial lifestyle can only be regained by semi-aquatic mammals, while a species with stronger aquatic adaptations (A2, A3) cannot regain terrestriality (figure 2*a* and table 1; electronic supplementary material, figure S2). The mean rate matrix obtained from model averaging and thus incorporating model uncertainty also captures this pattern, with transition rates from A3 to A2 and A2 to A1 being near-zero (electronic supplementary material, table S2). The median number of transitions estimated across the mammalian phylogeny is greater between A0 and A1, with 37 A0 → 1 and 22 A1 → 0 transitions, than between A1 and A2, and A2 and A3 (figure 2*b*).

**Table 1.** Mean ΔAICc scores and Akaike weights (AICwt)—and their standard deviation shown in parentheses—for the seven different models tested. The ‘IR_2132_’ model (electronic supplementary material, figure S1e) was the best one across all trees.

	ER	SYM	ORD	IR_32_	IR_2132_	IR_102132_	ARD
Mean ΔAICc	67.084 (15.320)	20.087 (14.045)	4.008 (10.253)	2.004 (10.253)	0.000 (10.253)	21.466 (14.861)	10.252 (10.324)
Mean AICwt	0.000 (0.000)	0.001 (0.005)	0.089 (0.001)	0.243 (0.003)	0.661 (0.009)	0.001 (0.008)	0.004 (0.001)

When estimating the rates of transition across different clades, we found that Carnivora, Rodentia, Didelphimorphia, Afrosoricida, Eulipotyphla, Cetartiodactyla and Monotremata had the highest A0 → 1 rates. Carnivora also has the highest transition rates from A1 to A2 compared to Sirenia and Cetartiodactyla (electronic supplementary material, table S3).

### (b) Aquatic adaptations and body mass and dietary changes

**Figure 3.**
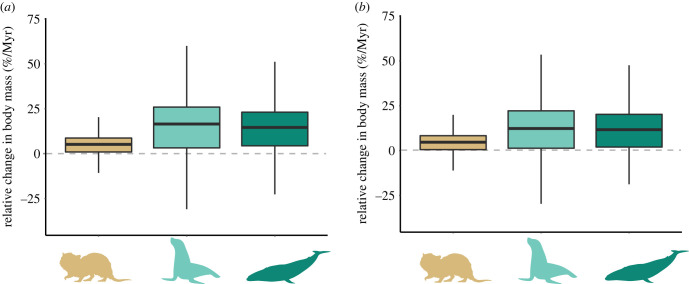
Relative change in body mass per Myr, indicating increase in body mass in semi-aquatic and fully aquatic mammals: (*a*) without hyperprior on *µ*_0_; (*b*) adding hyperprior on *µ*_0_. Coloured silhouettes represent the aquatic categories: A1, A2 and A3, respectively.

We found support for a consistent evolution of greater body mass in semi-aquatic and aquatic lineages. The posterior probabilities of a positive trend in body mass evolution ranged from 0.79 for semi-aquatic mammals (A1), to 0.87 for A3 (table 2). The trend was weaker for A1 lineages and stronger for more water-adapted categories (figure 3). For lineages classified as A1, we estimated a relative body mass increase of 4.66%/Myr, while for lineages classified as A2 and A3 the trend was three times steeper, with a relative body mass increase of 12.32 and 11.67%/Myr, respectively. The results did not change substantially using different priors on the trend parameters or when using different constraints on the trend parameters across aquatic classes (see electronic supplementary material, tables S5–S8, for comparison).

**Table 2.** Mean and 95% credible interval for the trend values, and probability of positive trends among the three aquatic categories.

	no hyperprior	hyperprior
*P*(A1 > 0)	78.6%	79.5%
mean (A1)	0.015	0.015
95% CI (A1)	−0.026–0.052	−0.019–0.050
*P*(A2 > 0)	81.2%	81.6%
mean (A2)	0.046	0.041
95% CI (A2)	−0.065–0.138	−0.031–0.127
*P*(A3 > 0)	87.5%	84.3%
mean (A3)	0.047	0.060
95% CI (A3)	−0.023–0.116	−0.020–0.112

Our analysis combining aquatic adaptations with diet revealed a significantly positive correlation between herbivore diet and terrestrially (A0), indicating that herbivory is more common in terrestrial mammals. We also found significant or weakly significant (predictive *p*-values 0.00–0.07) positive correlations between carnivory and all three aquatic groups and negative correlations with herbivory (predictive *p*-values 0.97–1.00; electronic supplementary material, table S4). This indicates that mammal species with some degree of adaptations to aquatic environments are disproportionally likely to have a carnivorous diet.

### (c) Estimating trends with simulated data

**Figure 4.**
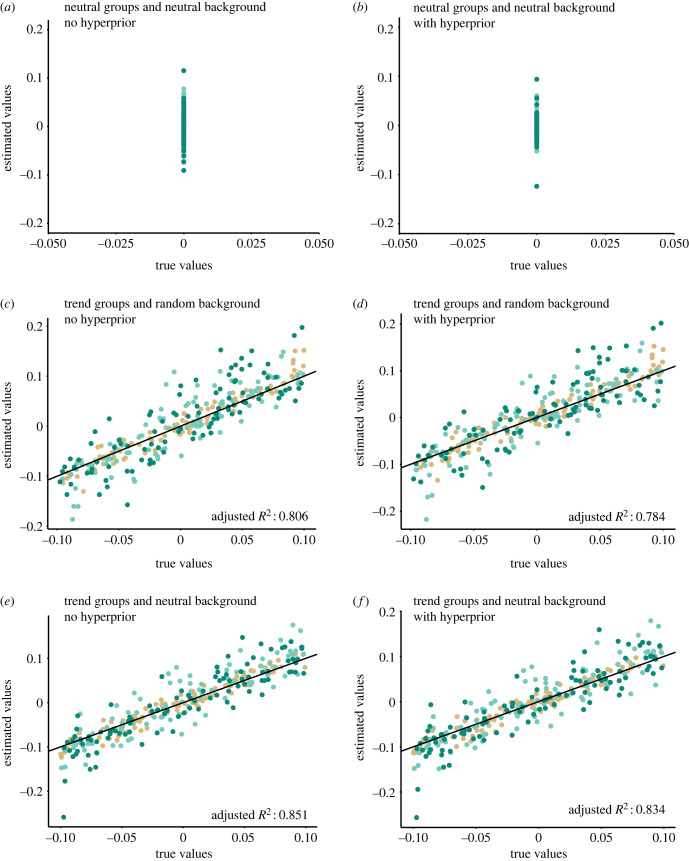
Estimated scenarios (*a*) with trends in the water-related groups and background (terrestrial groups) being neutral, without hyperprior; (*b*) the same scenario but adding hyperprior; (*c*) allowing each water-related group to have its own trend in body mass and terrestrial groups (background) being random; (*d*) same scenario but adding the hyperprior; (*e*) allowing each water-related group to have its own trend in body mass but terrestrial groups (background) are neutral; (*f*) same scenario but with hyperprior. Coloured points represent the aquatic categories: light brown = A1, light green = A2, dark green = A3. The black line represents the 1–1 diagonal, representing the ‘true values’ (*x*-axis).

Our simulations show high accuracy and low false positive rates (less than 5%), which decreased further when using a hyperprior on the trend parameter (*μ*) (table 3 and figure 4). The addition of the hyperprior resulted in higher accuracy, particularly in the ‘neutral groups and neutral background’ scenario. Without hyperprior, the absence of trends (true negative) was correctly estimated in 91–97% of the cases, while the same scenario with hyperprior the true negatives were 97–100% of the cases (table 3 and figure 4*a*,*b*). In simulations with non-zero trends, the true positive rates ranged between 84 and 94% and the presence of non-neutral background evolution in the A0 lineages (scenario 3) did not alter substantially the accuracy and robustness of the trends inferred for lineages in A1–3 (table 3; figure 4). Overall, the results show that the simulated trends in the A1–3 clades were reliably identifiable and that the use of a hyperprior generally makes the trend estimates more conservative, reducing the false positive rates.

**Table 3.** Three simulated scenarios without and with hyperprior on *µ*_0_, showing the accuracy and high coverage in the estimated results.

	group	neutral groups (A1, A2, A3) and neutral background (A0)			trend groups (A1, A2, A3) and random background (A0)	trend groups (A1, A2, A3) and neutral background (A0)
		underestimate	true negative	overestimate	true positive	true positive
**hyperprior**						
	A1	0.01	0.97	0.02	0.94	0.95
	A2	–	1.00	–	0.94	0.92
	A3	–	0.98	0.02	0.87	0.85
**no hyperprior**						
	A1	0.05	0.92	0.03	0.94	0.92
	A2	0.01	0.97	0.02	0.92	0.94
	A3	0.04	0.91	0.05	0.84	0.88

## 4. Discussion

### (a) Dollo's Law in marine mammals

In the history of vertebrates, water-to-land transition is thought to have happened only once, while the opposite transition occurred several times [1,14,55]. Our analyses show a similar pattern within mammals, where evolution to aquatic environments occurred in several lineages, and transitions to fully aquatic life were inferred to be irreversible. Yet, when considering intermediate adaptations, we also found that some semi-aquatic transitions are reversible. Our analyses identify the presence of a threshold, between our categories A1 and A2, after which aquatic adaptations become irreversible.

A secondary aquatic lifestyle typically involves several adaptations, enhancing species swimming ability, but also changing sensory system, reproduction, feeding strategies, and lung capacity, in both living and extinct species [8,15,56]. Some stem cetaceans (Archaeoceti), for example, exhibited a more semi-aquatic lifestyle, in line with the predictions of our estimated model, implying sequential evolution from A0 to A3. Their adaptations mainly involved their locomotory and sensory systems [57,58]. Similarly, stem sirenians displayed quadrupedal locomotion and had an amphibious lifestyle, again demonstrating the presence of step-wise evolution into fully aquatic forms, evolving the first fully aquatic forms by the end of Eocene [59]. Stem pinnipeds, from Oligocene, displayed paddle-like limbs, but yet relying more on terrestrial environments than extant pinnipeds [60].

Dollo's Law postulates that once a complex trait is lost, it cannot be regained [16,18,61,62]. The law is also thought to be generally true in cases such as tooth loss [22,55,56] (but see [60]), or loss of ability to fly in birds [18,64]. While limb reduction among different lineages of tetrapods (e.g. whales, squamates, birds and amphibians) can be interpreted as the loss of a complex trait, the irreversibility of such reduction remains debated [65–68]. The irreversibility of aquatic adaptations can be attributed to different non-exclusive mechanisms. Intrinsic mechanisms are linked to release of selective pressure on non-expressed genes and accumulation of deleterious mutations. For instance, hind-limb reduction in tetrapods is related to multiple gene expression loss, including *Sonic hedgehog* (*Shh*), which gradually became unexpressed in the Cetacean lineage [69,70]. Adaptive changes in the *Homeobox* (*Hox*) gene expression and in its regulation underwent convergent evolution in fully aquatic mammals (whales, pinnipeds and sirenians) along with several morphological and physiological adaptations to aquatic lifestyle [13,71–75]. The strong genomic implications linked to adaptations to a fully aquatic life are likely instrumental to making this transition irreversible.

Extrinsic mechanisms are also likely to play a role in determining the irreversibility of aquatic adaptation in mammals. Aquatic lineages underwent multiple morphological adaptations including, along with limb reduction, the evolution of different feeding modes. This is reflected in their distinct cranial morphology, allowing them to exploit different niches throughout their transition from land to water [70,71,76,77] and achieving larger body sizes, as a response of their energetic demands [11,24]. After that transition, competition with incumbent terrestrial carnivores, which can use their limbs for hunting more effectively on land and manipulate their preys [78,79], may have prevented any possibility for a reversal.

### (b) Bergmann's rule and aquatic transitions

The evolution of mammalian body mass patterns has been widely discussed in the literature showing a wide range of patterns and large heterogeneities across clades [11,80–85]. Our analyses indeed detected substantial variation in evolutionary rates among mammalian lineages, thus providing further evidence that changes in body mass follow a highly heterogeneous process. Within this variation, however, we also found that body mass consistently increases in lineages as they transitioned to aquatic environments.

Along with other adaptations, such as limb reduction, changes in sensorial systems, and diet, increased body mass in semi-aquatic and fully aquatic mammals can be interpreted in the light of different hypotheses, as a result of the land-to-water transition. First, terrestrial and aquatic environments impose different body size constraints related to locomotion, habitat and food availability [86]. Aquatic realms can relax some of these constraints, allowing larger body sizes due to neutral buoyance [87] and providing access to larger amounts of protein, sustaining more and larger carnivores [86]. For instance, the evolution of filter feeding and lunge feeding enabled even larger sizes in cetaceans [11,86]. However, aquatic environments also impose new constraints especially related to thermoregulation, limiting minimum body sizes in water due to higher rate of heat loss [27,88].

Studies have found support for Bergmann's rule in aquatic mammals (A2 and A3), in agreement with the heat conservation hypothesis (larger animals have lower surface–volume ratio, thus, lower heat loss, allowing them to endure cold temperatures and in water realm with high thermal conductivity compared to terrestrial one) [27,89,90]. When transitioning to aquatic lifestyles, internal insulation through the blubber layer became more effective compared to fur, with the additional benefit of helping in buoyance control [27,30,91]. A similar pattern likely applies to birds with strong aquatic adaptations, like the penguins, where the blubber layer serves for both thermoregulation and buoyance [92,93]. Several penguins also evolved to very large size including the extant emperor penguin and other even larger extinct taxa [94]. It is difficult, however, to compare size evolution in penguins to mammals because flight ability poses an upper maximum body size in flying birds [95] and penguins may have become larger as a consequence of a release from that selective pressure.

Lineages classified as A2 have retained both terrestrial and aquatic thermic insulation adaptations, fur (water-repellent) and blubber layer, respectively—except for the sea otter (*Enhydra lutris*), which does not possess blubber but exhibits exceptionally dense fur—as they should be able to maintain thermal balance in both realms [21], thus possibly limiting their maximum body sizes. Nevertheless, large body sizes are related to the use of internal insulation as primary heat source, as large pinnipeds tend to rely more on blubber than fur insulation compared to smaller ones [96].

### (c) Links between carnivory and land-to-water transitions

We found evidence for a strong association between carnivory (defined here as lineages feeding on vertebrates or invertebrates) and adaptations to aquatic environments. Our finding supports the hypothesis that food resources in water are an important factor determining aquatic colonization, especially for semi-aquatic species [1,43]. Previous observation further showed that among the few semi-aquatic rodents, at least half of them were carnivores and showed larger body mass [86], indicating that this association is present even in semi-aquatic groups.

Food availability may not readily explain the scarcity of aquatic herbivores. There are many resources available in the seagrasses and algal beds on low water marine settings. Interestingly, these are generally not useable by ectothermic vertebrates in colder water, likely because enzymes needed to break down plant or algal material require higher temperatures [97]. We could therefore expect herbivorous endotherms to be common at higher latitudes, similar to the reverse latitudinal gradient seen in fully marine ectotherms [98]. The increased heat loss in aquatic environments may however require a faster metabolism and therefore a more nutritious diet. Therefore, it may be not a coincidence that sirenians, an exception as a fully marine fully herbivorous taxa, consistently have a large body size [33]. In that regard, it is further striking that Steller's sea cow, which until its recent human-driven extinction was the only cold-water species in this group, was about ten times heavier than the extant species.

## 5. Conclusion

Aquatic adaptations in mammals independently evolved to different degrees in several lineages. Through the discretization of this spectrum of adaptation to aquatic environments we found that transitions between terrestrial and semi-aquatic lifestyles occurred multiple times in both directions, while these transitions become rarer and irreversible in more strictly water-adapted lineages. While the causal relationships and relative timing of the emergence of different adaptive traits remain difficult to assess, aquatic adaptations in mammals are consistently associated with relatively larger body sizes, possibly due to thermoregulation constraints following Bergmann's rule, and a more carnivorous diet. The application of our analytical framework beyond mammals can clarify the generality of the history of aquatic transitions across other animal clades.

## Data Availability

The data and scripts used in this study are available in the Dryad Digital Repository [99]. Supplementary figures and tables are provided in electronic supplementary material. Additional information is provided in electronic supplementary material [100].

## References

[1] Vermeij GJ, Dudley R. 2000 Why are there so few evolutionary transitions between aquatic and terrestrial ecosystems? Biol. J. Linn. Soc. **70**, 541-554. (10.1111/j.1095-8312.2000.tb00216.x)

[2] Vermeij GJ, Motani R. 2018 Land to sea transitions in vertebrates: the dynamics of colonization. Paleobiology **44**, 237-250. (10.1017/pab.2017.37)

[3] Benson RBJ, Butler RJ. 2011 Uncovering the diversification history of marine tetrapods: ecology influences the effect of geological sampling biases. Geol. Soc. Lond. Spec. Publ. **358**, 191-208. (10.1144/SP358.13)

[4] Benson RBJ, Druckenmiller PS. 2014 Faunal turnover of marine tetrapods during the Jurassic–Cretaceous transition. Biol. Rev. **89**, 1-23. (10.1111/brv.12038) 23581455

[5] Kelley NP, Pyenson ND. 2015 Evolutionary innovation and ecology in marine tetrapods from the Triassic to the Anthropocene. Science **348**, aaa3716. (10.1126/science.aaa3716) 25883362

[6] Massare JA. 1987 Tooth morphology and prey preference of Mesozoic marine reptiles. J. Vertebr. Paleontol. **7**, 121-137. (10.1080/02724634.1987.10011647)

[7] Motani R, Chen Xh, Jiang Dy, Cheng L, Tintori A, Rieppel O. 2015 Lunge feeding in early marine reptiles and fast evolution of marine tetrapod feeding guilds. Sci. Rep. **5**, 8900. (10.1038/srep08900) 25754468 PMC4354009

[8] Uhen MD. 2007 Evolution of marine mammals: back to the sea after 300 million years. Anat. Rec. **290**, 514-522. (10.1002/ar.20545) 17516441

[9] Lindgren J, Caldwell MW, Konishi T, Chiappe LM. 2010 Convergent evolution in aquatic tetrapods: insights from an exceptional fossil mosasaur. PLoS ONE **5**, e11998. (10.1371/journal.pone.0011998) 20711249 PMC2918493

[10] Taylor MA. 2000 Functional significance of bone ballastin in the evolution of buoyancy control strategies by aquatic tetrapods. Hist. Biol. **14**, 15-31. (10.1080/10292380009380550)

[11] Gearty W, McClain CR, Payne JL. 2018 Energetic tradeoffs control the size distribution of aquatic mammals. Proc. Natl. Acad. Sci. USA **115**, 4194-4199. (10.1073/pnas.1712629115) 29581289 PMC5910812

[12] Pyenson ND, Kelley NP, Parham JF. 2014 Marine tetrapod macroevolution: physical and biological drivers on 250 Ma of invasions and evolution in ocean ecosystems. Palaeogeogr. Palaeoclimatol. Palaeoecol. **400**, 1-8. (10.1016/j.palaeo.2014.02.018)

[13] Figueirido B, Serrano FJ, Pérez-Ramos A, Esteban JM, Ferrón HG, Martín-Serra A. 2022 Body-axis organization in tetrapods: a model-system to disentangle the developmental origins of convergent evolution in deep time. Biol. Lett. **18**, 20220047. (10.1098/rsbl.2022.0047) 35382583 PMC8984341

[14] Gutarra S, Rahman IA. 2022 The locomotion of extinct secondarily aquatic tetrapods. Biol. Rev. **97**, 67-98. (10.1111/brv.12790) 34486794

[15] Reidenberg JS. 2007 Anatomical adaptations of aquatic mammals. Anat. Rec. **290**, 507-513. (10.1002/ar.20541) 17516440

[16] Simpson GG. 1953 The major features of evolution. New York, NY: Columbia University Press.

[17] Aboitiz F. 1990 Behavior, body types and the irreversibility of evolution. Acta Biotheor. **38**, 91-101. (10.1007/BF00047546) 2113334

[18] Collin R, Miglietta MP. 2008 Reversing opinions on Dollo's Law. Trends Ecol. Evol. **23**, 602-609. (10.1016/j.tree.2008.06.013) 18814933

[19] Pyenson ND, Vermeij GJ. 2016 The rise of ocean giants: maximum body size in Cenozoic marine mammals as an indicator for productivity in the Pacific and Atlantic Oceans. Biol. Lett. **12**, 20160186. (10.1098/rsbl.2016.0186) 27381883 PMC4971165

[20] Goldbogen JA, Madsen PT. 2018 The evolution of foraging capacity and gigantism in cetaceans. J. Exp. Biol. **221**, jeb166033. (10.1242/jeb.166033) 29895582

[21] Favilla AB, Horning M, Costa DP. 2022 Advances in thermal physiology of diving marine mammals: the dual role of peripheral perfusion. Temperature **9**, 46-66. (10.1080/23328940.2021.1988817) PMC9154795 35655662

[22] IUCN. 2022 The IUCN Red List of Threatened Species. See https://www.iucnredlist.org/en (accessed 19 November 2022).

[23] Veron G, Patterson BD, Reeves R. 2008 Global diversity of mammals (Mammalia) in freshwater. In Freshwater animal diversity assessment (eds EV Balian, C Lévêque, H Segers, K Martens), pp. 607-617. Dordrecht, The Netherlands: Springer.

[24] Goldbogen JA et al. 2019 Why whales are big but not bigger: physiological drivers and ecological limits in the age of ocean giants. Science **366**, 1367-1372. (10.1126/science.aax9044) 31831666

[25] Bergmann C. 1847 Ober die Verhaltnisse der Warmeokonomie der Thiere zu ihrer Grosse. Gottinger Studien **3**, 595-708.

[26] Meiri S. 2011 Bergmann's rule: what's in a name? Glob. Ecol. Biogeogr. **20**, 203-207. (10.1111/j.1466-8238.2010.00577.x)

[27] Ahlborn BK, Blake RW. 1999 Lower size limit of aquatic mammals. Am. J. Phys. **67**, 920-922. (10.1119/1.19150)

[28] McHuron EA et al. 2022 Key questions in marine mammal bioenergetics. Conserv. Physiol. **10**, coac055. (10.1093/conphys/coac055) 35949259 PMC9358695

[29] Lemell P, Natchev N, Beisser CJ, Heiss E. 2019 Feeding in turtles: understanding terrestrial and aquatic feeding in a diverse but monophyletic group. In Feeding in vertebrates: evolution, morphology, behavior, biomechanics (eds V Bels, IQ Whishaw), pp. 611-642. Cham, Switzerland: Springer International.

[30] Fish FE. 2000 Biomechanics and energetics in aquatic and semiaquatic mammals: platypus to whale. Physiol. Biochem. Zool. **73**, 683-698. (10.1086/318108) 11121343

[31] Gearty W, Payne JL. 2020 Physiological constraints on body size distributions in Crocodyliformes. Evolution **74**, 245-255. (10.1111/evo.13901) 31943148

[32] Gearty W, Carrillo E, Payne JL. 2021 Ecological filtering and exaptation in the evolution of marine snakes. Am. Nat. **198**, 506-521. (10.1086/716015) 34559607

[33] Faurby S, Davis M, Pedersen RØ, Schowanek SD, Antonelli A, Svenning J. 2018 PHYLACINE 1.2: the phylogenetic atlas of mammal macroecology. Ecology **99**, 2626. (10.1002/ecy.2443) 29989146

[34] Smith FA, Lyons SK, Ernest SKM, Jones KE, Kaufman DM, Dayan T, Marquet PA, Brown JH, Haskell JP. 2003 Body mass of late quaternary mammals. Ecology **84**, 3403. (10.1890/02-9003)

[35] Faurby S, Svenning JC. 2016 Resurrection of the Island Rule: human-driven extinctions have obscured a basic evolutionary pattern. Am. Nat. **187**, 812-820. (10.1086/686268) 27172600

[36] Wilman H, Belmaker J, Simpson J, de la Rosa C, Rivadeneira MM, Jetz W. 2014 EltonTraits 1.0: species-level foraging attributes of the world's birds and mammals. Ecology **95**, 2027. (10.1890/13-1917.1)

[37] Kissling WD, Dalby L, Fløjgaard C, Lenoir J, Sandel B, Sandom C, Trøjelsgaard K, Svenning J-C. 2014 Establishing macroecological trait datasets: digitalization, extrapolation, and validation of diet preferences in terrestrial mammals worldwide. Ecol. Evol. **4**, 2913-2930. (10.1002/ece3.1136) 25165528 PMC4130448

[38] Upham NS, Esselstyn JA, Jetz W. 2019 Inferring the mammal tree: species-level sets of phylogenies for questions in ecology, evolution, and conservation. PLoS Biol. **17**, e3000494. (10.1371/journal.pbio.3000494) 31800571 PMC6892540

[39] Mittermeier RA, Wilson DE. 2014 Handbook of the mammals of the world: vol. 4. Sea mammals. Barcelona, Spain: Lynx Edicions.

[40] Lacher TE, Mittermeier RA, Wilson DE. 2016 Handbook of the mammals of the world: vol. 6. Lagomorphs and rodents I. Barcelona, Spain: Lynx Edicions.

[41] Wilson DE, Mittermeier RA. 2018 Handbook of the mammals of the world: vol. 8. Insectivores, sloths and colugos. Barcelona, Spain: Lynx Edicions.

[42] Wilson DE, Lacher TE, Mittermeier RA. 2017 Handbook of the mammals of the world: vol. 7. Rodents II. Barcelona, Spain: Lynx Edicions.

[43] Motani R, Vermeij GJ. 2021 Ecophysiological steps of marine adaptation in extant and extinct non-avian tetrapods. Biol. Rev. **96**, 1769-1798. (10.1111/brv.12724) 33904243

[44] Harmon LJ. 2019 *Phylogenetic comparative methods*.

[45] R Core Team. 2021 R: a language and environment for statistical computing. Vienna, Austria: R Foundation for Statistical Computing.

[46] Beaulieu JM, O'Meara BC, Donoghue MJ. 2013 Identifying hidden rate changes in the evolution of a binary morphological character: the evolution of plant habit in Campanulid angiosperms. Syst. Biol. **62**, 725-737. (10.1093/sysbio/syt034) 23676760

[47] FitzJohn RG, Maddison WP, Otto SP. 2009 Estimating trait-dependent speciation and extinction rates from incompletely resolved phylogenies. Syst. Biol. **58**, 595-611. (10.1093/sysbio/syp067) 20525612

[48] Wagenmakers EJ, Farrell S. 2004 AIC model selection using Akaike weights. Psychon. Bull. Rev. **11**, 192-196. (10.3758/BF03206482) 15117008

[49] Revell LJ. 2012 phytools: an R package for phylogenetic comparative biology (and other things). Methods Ecol. Evol. **3**, 217-223. (10.1111/j.2041-210X.2011.00169.x)

[50] Huelsenbeck JP, Nielsen R, Bollback JP. 2003 Stochastic mapping of morphological characters. Syst. Biol. **52**, 131-158. (10.1080/10635150390192780) 12746144

[51] Bollback JP. 2006 SIMMAP: stochastic character mapping of discrete traits on phylogenies. BMC Bioinf. **7**, 88. (10.1186/1471-2105-7-88) PMC1403802 16504105

[52] Silvestro D et al. 2019 Early arrival and climatically-linked geographic expansion of New World monkeys from tiny African ancestors. Syst. Biol. **68**, 78-92. (10.1093/sysbio/syy046) 29931325 PMC6292484

[53] Rolland J, Silvestro D, Schluter D, Guisan A, Broennimann O, Salamin N. 2018 The impact of endothermy on the climatic niche evolution and the distribution of vertebrate diversity. Nat. Ecol. Evol. **2**, 459-464. (10.1038/s41559-017-0451-9) 29379185

[54] Flannery-Sutherland JT, Silvestro D, Benton MJ. 2022 Global diversity dynamics in the fossil record are regionally heterogeneous. Nat. Commun. **13**, 2751. (10.1038/s41467-022-30507-0) 35585069 PMC9117201

[55] Laurin M, Girondot M, de Ricqlès A. 2000 Early tetrapod evolution. Trends Ecol. Evol. **15**, 118-123. (10.1016/S0169-5347(99)01780-2) 10675932

[56] Fordyce RE. 1980 Whale evolution and Oligocene southern ocean environments. Palaeogeogr. Palaeoclimatol. Palaeoecol. **31**, 319-336. (10.1016/0031-0182(80)90024-3)

[57] Fordyce RE, Marx FG. 2018 Gigantism precedes filter feeding in baleen whale evolution. Curr. Biol. **28**, 1670-1676. e2. (10.1016/j.cub.2018.04.027) 29754903

[58] Nummela S, Thewissen JGM, Bajpai S, Hussain ST, Kumar K. 2004 Eocene evolution of whale hearing. Nature **430**, 776-778. (10.1038/nature02720) 15306808

[59] Domning DP. 2018 Sirenian evolution. In Encyclopedia of marine mammals, 3rd edn (eds B Würsig, JGM Thewissen, KM Kovacs), pp. 856-859. New York, NY: Academic Press.

[60] Berta A, Churchill M, Boessenecker RW. 2018 The origin and evolutionary biology of pinnipeds: seals, sea lions, and walruses. Annu. Rev. Earth Planet. Sci. **46**, 203-228. (10.1146/annurev-earth-082517-010009)

[61] Dollo L. 1893 Les lois de l’évolution. Bull. Soc. Belge. Geol. Pal. Hydr. **VII**, 164-166.

[62] Gould SJ. 1970 Dollo on Dollo's Law: irreversibility and the status of evolutionary laws. J. Hist. Biol. **3**, 189-212. (10.1007/BF00137351) 11609651

[63] Paluh DJ, Dillard WA, Stanley EL, Fraser GJ, Blackburn DC. 2021 Re-evaluating the morphological evidence for the re-evolution of lost mandibular teeth in frogs. Evolution **75**, 3203-3213. (10.1111/evo.14379) 34674263 PMC9299036

[64] Maderspacher F. 2017 Evolution: flight of the ratites. Curr. Biol. **27**, R110-R113. (10.1016/j.cub.2016.12.023) 28171755

[65] Bergmann PJ, Morinaga G. 2019 The convergent evolution of snake-like forms by divergent evolutionary pathways in squamate reptiles. Evolution **73**, 481-496. (10.1111/evo.13651) 30460998

[66] Camaiti M, Evans AR, Hipsley CA, Chapple DG. 2021 A farewell to arms and legs: a review of limb reduction in squamates. Biol. Rev. **96**, 1035-1050. (10.1111/brv.12690) 33538028

[67] Galis F, Arntzen JW, Lande R. 2010 Dollo's Law and the irreversibility of digit loss in Bachia. Evolution **64**, 2466-2476. 20500218 10.1111/j.1558-5646.2010.01041.x

[68] Kohlsdorf T, Wagner GP. 2006 Evidence for the reversibility of digit loss: a phylogenetic study of limb evolution in Bachia (Gymnophthalmidae: Squamata). Evolution **60**, 1896-1912. 17089974

[69] Sagai T, Hosoya M, Mizushina Y, Tamura M, Shiroishi T. 2005 Elimination of a long-range cis-regulatory module causes complete loss of limb-specific Shh expression and truncation of the mouse limb. Development **132**, 797-803. (10.1242/dev.01613) 15677727

[70] Thewissen JGM, Cohn MJ, Stevens LS, Bajpai S, Heyning J, Horton WE. 2006 Developmental basis for hind-limb loss in dolphins and origin of the cetacean bodyplan. Proc. Natl Acad. Sci. USA **103**, 8414-8418. (10.1073/pnas.0602920103) 16717186 PMC1482506

[71] Bejder L, Hall BK. 2002 Limbs in whales and limblessness in other vertebrates: mechanisms of evolutionary and developmental transformation and loss. Evol. Dev. **4**, 445-458. (10.1046/j.1525-142X.2002.02033.x) 12492145

[72] Foote AD et al. 2015 Convergent evolution of the genomes of marine mammals. Nat. Genet. **47**, 272-275. (10.1038/ng.3198) 25621460 PMC4644735

[73] Menke DB, Guenther C, Kingsley DM. 2008 Dual hindlimb control elements in the Tbx4 gene and region-specific control of bone size in vertebrate limbs. Development **135**, 2543-2553. (10.1242/dev.017384) 18579682

[74] Nery MF, Borges B, Dragalzew AC, Kohlsdorf T. 2016 Selection on different genes with equivalent functions: the convergence story told by Hox genes along the evolution of aquatic mammalian lineages. BMC Evol. Biol. **16**, 113. (10.1186/s12862-016-0682-4) 27209096 PMC4875654

[75] Petit F, Sears KE, Ahituv N. 2017 Limb development: a paradigm of gene regulation. Nat. Rev. Genet. **18**, 245-258. (10.1038/nrg.2016.167) 28163321

[76] Hocking DP, Marx FG, Park T, Fitzgerald EMG, Evans AR. 2017 Reply to comment by Kienle *et al*. 2017. Proc. R. Soc. B **284**, 20171836. (10.1098/rspb.2017.1836) PMC5627218 28954917

[77] Hocking DP, Marx FG, Park T, Fitzgerald EMG, Evans AR. 2017 A behavioural framework for the evolution of feeding in predatory aquatic mammals. Proc. R. Soc. B **284**, 20162750. (10.1098/rspb.2016.2750) PMC5360926 28250183

[78] Van Valkenburgh B. 1985 Locomotor diversity within past and present guilds of large predatory mammals. Paleobiology **11**, 406-428. (10.1017/S0094837300011702)

[79] Van Valkenburgh B. 2007 Déjà vu: the evolution of feeding morphologies in the Carnivora. Integr. Comp. Biol. **47**, 147-163. (10.1093/icb/icm016) 21672827

[80] Alroy J. 1998 Cope's Rule and the dynamics of body mass evolution in North American fossil mammals. Science **280**, 731-734. (10.1126/science.280.5364.731) 9563948

[81] Cooper N, Purvis A. 2010 Body size evolution in mammals: complexity in tempo and mode. Am. Nat. **175**, 727-738. (10.1086/652466) 20394498

[82] Slater GJ. 2013 Phylogenetic evidence for a shift in the mode of mammalian body size evolution at the Cretaceous-Palaeogene boundary. Methods Ecol. Evol. **4**, 734-744. (10.1111/2041-210X.12084)

[83] Smith FA et al. 2010 The evolution of maximum body size of terrestrial mammals. Science **330**, 1216-1219. (10.1126/science.1194830) 21109666

[84] Smith FA, Elliott Smith RE, Lyons SK, Payne JL. 2018 Body size downgrading of mammals over the late quaternary. Science **360**, 310-313. (10.1126/science.aao5987) 29674591

[85] Venditti C, Meade A, Pagel M. 2011 Multiple routes to mammalian diversity. Nature **479**, 393-396. (10.1038/nature10516) 22012260

[86] Price SA, Hopkins SSB. 2015 The macroevolutionary relationship between diet and body mass across mammals. Biol. J. Linn. Soc. **115**, 173-184. (10.1111/bij.12495)

[87] Hays GC, Metcalfe JD, Walne AW. 2004 The implications of lung-regulated buoyancy control for dive depth and duration. Ecology **85**, 1137-1145. (10.1890/03-0251)

[88] Downhower JF, Blumer LS. 1988 Calculating just how small a whale can be. Nature **335**, 675. (10.1038/335675b0)3173490

[89] Adamczak SK, Pabst DA, McLellan WA, Thorne LH. 2020 Do bigger bodies require bigger radiators? Insights into thermal ecology from closely related marine mammal species and implications for ecogeographic rules. J. Biogeogr. **47**, 1193-1206. (10.1111/jbi.13796)

[90] Torres-Romero EJ, Morales-Castilla I, Olalla-Tárraga MÁ. 2016 Bergmann's rule in the oceans? Temperature strongly correlates with global interspecific patterns of body size in marine mammals. Global Ecol. Biogeogr. **25**, 1206-1215. (10.1111/geb.12476)

[91] Yuan Y et al. 2021 Comparative genomics provides insights into the aquatic adaptations of mammals. Proc. Natl Acad. Sci. USA **118**, e2106080118. (10.1073/pnas.2106080118) 34503999 PMC8449357

[92] Hind AT, Gurney WS. 1997 The metabolic cost of swimming in marine homeotherms. J. Exp. Biol. **200**, 531-542. (10.1242/jeb.200.3.531) 9057309

[93] Taylor MA. 1994 Stone, bone or blubber? Buoyancy control. Mech. Physiol. Anim. Swimming **52**, 151. (10.1017/CBO9780511983641.012)

[94] Mayr G, Scofield RP, De Pietri VL, Tennyson AJD. 2017 A Paleocene penguin from New Zealand substantiates multiple origins of gigantism in fossil Sphenisciformes. Nat. Commun. **8**, 1927. (10.1038/s41467-017-01959-6) 29233963 PMC5727159

[95] Maurer BA. 1998 The evolution of body size in birds. I. Evidence for non-random diversification. Evol. Ecol. **12**, 925-934. (10.1023/A:1006512121434)

[96] Liwanag HEM, Berta A, Costa DP, Budge SM, Williams TM. 2012 Morphological and thermal properties of mammalian insulation: the evolutionary transition to blubber in pinnipeds. Biol. J. Linn. Soc. **107**, 774-787. (10.1111/j.1095-8312.2012.01992.x)

[97] Vejříková I et al. 2016 Distribution of herbivorous fish is frozen by low temperature. Sci. Rep. **6**, 39600. (10.1038/srep39600) 28004804 PMC5177937

[98] Grady JM et al. 2019 Metabolic asymmetry and the global diversity of marine predators. Science **363**, eaat4220. (10.1126/science.aat4220) 30679341

[99] Farina B, Faurby S, Silvestro D. 2023 Data from: Dollo meets Bergmann: morphological evolution in secondary aquatic mammals. Dryad Digital Repository. (10.5061/dryad.pc866t1v5) PMC10336382 37434524

[100] Farina BM, Faurby S, Silvestro D. 2023 Dollo meets Bergmann: morphological evolution in secondary aquatic mammals. Figshare. (10.6084/m9.figshare.c.6707592) PMC10336382 37434524

